# An Assessment of the Impact of Coronavirus Disease (COVID-19) Pandemic on National Antimicrobial Consumption in Jordan

**DOI:** 10.3390/antibiotics10060690

**Published:** 2021-06-09

**Authors:** Sayer Al-Azzam, Nizar Mahmoud Mhaidat, Hayaa A. Banat, Mohammad Alfaour, Dana Samih Ahmad, Arno Muller, Adi Al-Nuseirat, Elizabeth A. Lattyak, Barbara R. Conway, Mamoon A. Aldeyab

**Affiliations:** 1 Clinical Pharmacy Department, Jordan University of Science and Technology, Irbid 22110, Jordan; salazzam@just.edu.jo (S.A.-A.); nizarm@just.edu.jo (N.M.M.); 2 Jordan Food and Drug Administration (JFDA), Amman 11181, Jordan; hayaa.banat@jfda.jo (H.A.B.); Mohammad.alfaour@jfda.jo (M.A.); danadawlah@gmail.com (D.S.A.); 3 Antimicrobial Resistance Division, World Health Organization, Avenue Appia 20, 1211 Geneva, Switzerland; amuller@who.int; 4 World Health Organization Regional Office for the Eastern Mediterranean, Cairo 11371, Egypt; nuseirata@who.int; 5 Scientific Computing Associates Corp., River Forest, IL 60305, USA; elizabeth.lattyak@sbcglobal.net; 6 Department of Pharmacy, School of Applied Sciences, University of Huddersfield, Huddersfield HD1 3DH, UK; b.r.conway@hud.ac.uk; 7 Institute of Skin Integrity and Infection Prevention, University of Huddersfield, Huddersfield HD1 3DH, UK

**Keywords:** antimicrobial consumption, antimicrobial resistance, AWaRe classification, drug utilization, antimicrobial stewardship

## Abstract

Coronavirus disease 2019 (COVID-19) has overlapping clinical characteristics with bacterial respiratory tract infection, leading to the prescription of potentially unnecessary antibiotics. This study aimed at measuring changes and patterns of national antimicrobial use for one year preceding and one year during the COVID-19 pandemic. Annual national antimicrobial consumption for 2019 and 2020 was obtained from the Jordan Food and Drug Administration (JFDA) following the WHO surveillance methods. The WHO Access, Watch, and Reserve (AWaRe) classification was used. Total antibiotic consumption in 2020 (26.8 DDD per 1000 inhabitants per day) decreased by 5.5% compared to 2019 (28.4 DDD per 1000 inhabitants per day). There was an increase in the use of several antibiotics during 2020 compared with 2019 (third generation cephalosporins (19%), carbapenems (52%), macrolides (57%), and lincosamides (106%)). In 2020, there was a marked reduction in amoxicillin use (−53%), while the use of azithromycin increased by 74%. National antimicrobial consumption of the Access group decreased by 18% from 2019 to 2020 (59.1% vs. 48.1% of total consumption). The use of the Watch group increased in 2020 by 26%. The study highlighted an increase in the use of certain antibiotics during the pandemic period that are known to be associated with increasing resistance. Efforts to enhance national antimicrobial stewardship are needed to ensure rational use of antimicrobials.

## 1. Introduction

The emergence and spread of multidrug-resistant bacteria and the decreased investment in antibiotic development threaten many achievements of modern medicine and pose a significant global threat to public health [1]. A continued rise in antimicrobial resistance (AMR) would lead to increased deaths caused by drug-resistant infections from currently 700,000 to 10 million every year, resulting in projected costs as high as USD 100 trillion worldwide by 2050 [2]. The overuse and misuse of antimicrobials has increased the risk of emergence and spread of AMR globally [1,2]. Due to the selective pressure imposed by antibiotic agents over bacterial populations, antibiotic consumption has been clearly shown to contribute to the selection and spread of drug resistant microorganisms [3,4,5]. The rapid evolution and development of antibiotic resistance responsible for human infection, and the decrease in the number of new antibiotics approved, critically necessitate conserving the effectiveness of existing antibiotics [1]. Since antimicrobial use is one of the main drivers of antimicrobial resistance, surveillance and the optimization of antibiotic use is essential to control antimicrobial resistance and is one of the Global Action Plan (GAP) objectives on AMR and included in the WHO Global Antimicrobial Resistance and Use Surveillance System [6,7]. The WHO supported the establishment of antimicrobial consumption surveillance systems [7]. This is essential for evaluating amounts, patterns, and trends in antimicrobial use, performing benchmarking and risk adjustment, providing essential data for setting and implementing targets to improve antibiotic use and resistance, linking antibiotic use with resistance, and raising awareness among healthcare professionals, policy-makers, and the public about the need to address inappropriate use of antimicrobials, as well as its contribution to the development of AMR [7,8,9,10]. Measuring antimicrobial use is important for informing antimicrobial stewardship activities [10,11].

At the end of December 2019, several cases of acute respiratory syndrome were reported in Wuhan City, China. A novel coronavirus, called severe acute respiratory syndrome coronavirus 2 (SARS-CoV-2) was identified as the main causative agent. The disease is now referred to as coronavirus disease 2019 (COVID-19) [12,13]. The outbreak in Wuhan spread rapidly, causing large epidemics of the disease worldwide. On January 30th, 2020, the COVID-19 outbreak was declared a public health emergency of international concern (PHEIC). COVID-19 has overlapping clinical and radiological characteristics with bacterial respiratory tract infection, leading to the prescription of potentially unnecessary antibiotics. Effective diagnostic specimens for conducting mass testing and for accurate diagnosis of COVID-19 is critical and deemed necessary [14]. The inappropriate use of antibiotics during the COVID-19 pandemic may further worsen the situation in relation to antimicrobial resistance [15,16,17]. An assessment to determine changes in antimicrobial consumption due the COVID-19 outbreak is needed.

The aim of this study is to measure and determine the change and patterns of national antimicrobial use in Jordan one year before and one year during the coronavirus disease 2019 (COVID-19) pandemic, that is, to compare antimicrobial consumption data for 2019 with 2020.

## 2. Results

The total antibiotic consumption in 2019 was 28.4 defined daily dose (DDD) per 1000 inhabitants per day, of which 27.3 DDD per 1000 inhabitants per day represented oral use (96%) and 1.1 DDD per 1000 inhabitants per day represented parenteral use (4%). The total antibiotic consumption in 2020 was 26.8 DDD per 1000 inhabitants per day, of which 25.2 DDD per 1000 inhabitants per day represented oral use (94%) and 1.6 DDD per 1000 inhabitants per day represented parenteral use (6%). During 2020, total antimicrobial consumption decreased by 5.5% compared to consumption in 2019. Analysis of national antimicrobial consumption demonstrated different patterns of use between 2019 and 2020 (Table 1, Table 2 and Table 3). The most frequently used antibiotics in 2019 were penicillins with extended spectrum (29.574%), and combinations of penicillins, including beta-lactamase inhibitors (17.059%), macrolides (11.405%), and fluoroquinolones (9.669%). The most frequently used antibiotics in 2020 were combinations of penicillins, including beta-lactamase inhibitors (18.069%), macrolides (17.947%), penicillins with extended spectrum (13.930%), and fluoroquinolones (11.188%); Table 1. Whereas some of the use of antibiotics was shown to decrease in 2020 (e.g., penicillins with extended spectrum (−53%), and first-generation cephalosporins (−35%)), other antibiotics had an increase in use compared with 2019 (e.g., third-generation cephalosporins (19%), carbapenems (52%), macrolides (57%), and lincosamides (106%); Table 1).

**Figure 1:**
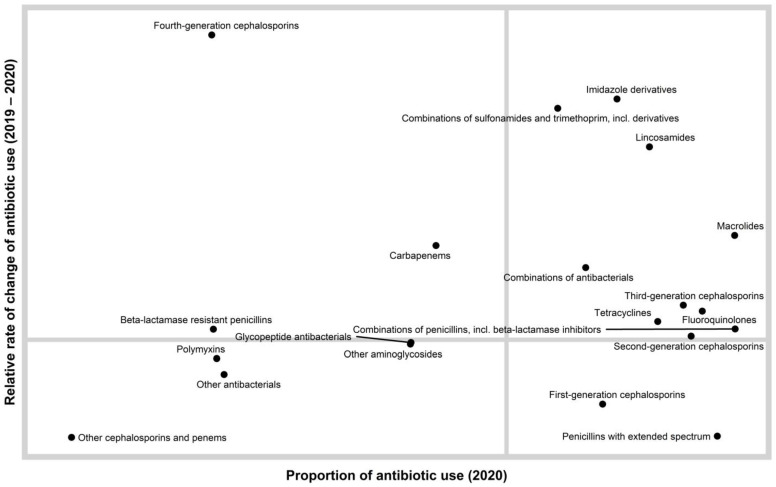
A quadrant chart showing the distribution of antibiotic classes according to rate of change of antibiotic use (2019–2020) and the proportion of antibiotic use in 2020. This chart enables a relative comparison of both antimicrobial levels and trends. We can consider the antimicrobials in the lower right quadrant as widely dispensed but at a lower rate than the previous year. We can consider the upper right quadrant as widely dispensed but at a higher rate than the previous year. The upper right quadrant contains antimicrobials that are candidates for further evaluation.

A quadrant chart showing the distribution of antibiotic classes according to relative rate of change of antibiotic use (2019–2020) and the proportion of antibiotic use in 2020 is presented in Figure 1.

In relation to specific antibiotics, a marked reduction in amoxicillin use was evident (2019 = 8.4 DDD per 1000 inhabitants per day, 2020 = 3.7 DDD per 1000 inhabitants per day; −53%), and the use of azithromycin increased in 2020 (74%; 2.5 DDD per 1000 inhabitants per day) compared to 2019 (1.5 DDD per 1000 inhabitants per day). The use of clindamycin in 2020 (1.19 DDD per 1000 inhabitants per day) increased compared with 2019 (0.43 DDD per 1000 inhabitants per day) by 192%. 

The use of hydroxychloroquine in 2020 (0.358 DDD per 1000 inhabitants per day) increased compared with 2019 (0.213 DDD per 1000 inhabitants per day) by 68.1%. According to WHO AWaRe classification (Access, Watch, and Reserve), and for 2019 and 2020, the percentage use of the Access group of national antimicrobial consumption was 59.13% and 48.67%, respectively. The latter represented a reduction in the use of antibiotics belonging to the Access group in 2020 compared with 2019 by 18% (Table 2). Use of the Watch group increased in 2020 by 26%, while the use of the Reserve group decreased in 2020 by 16% (Table 2). 

The most frequently used antibiotics, contributing to 90% of total national antibiotic use in 2019 and 2020, are presented in Table 3.

Analysis of national antiviral consumption showed different patterns of use between 2019 and 2020 (Table 4). The total antiviral use in 2019 and 2020 was 0.149 DDD per 1000 inhabitants per day and 0.205 DDD per 1000 inhabitants per day, respectively, representing a 37.7% increase in its use in 2020. There was a marked reduction (by 51%) in aciclovir use in 2020 (0.039 DDD per 1000 inhabitants per day), compared with 2019 (0.058 DDD per 1000 inhabitants per day). Favipiravir was not used in 2019 while its use represented 20% of all antiviral consumption in 2020 (0.04 DDD per 1000 inhabitants per day). The consumption of each antiviral agent for 2019 and 2020, expressed as DDD per 1000 inhabitants per day, and the change rate are presented in Table 4. 

**Figure 2:**
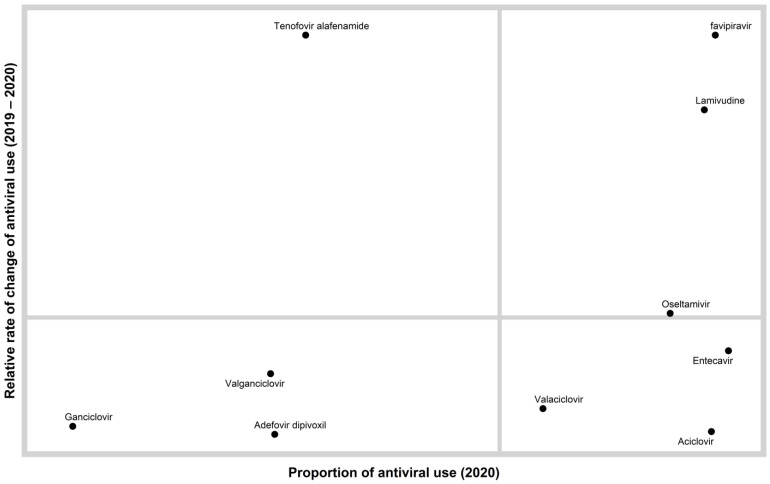
A quadrant chart showing the distribution of antiviral agents according to the rate of change of antiviral use (2019–2020), and the proportion of antiviral use in 2020. This chart enables a relative comparison of both antiviral levels and trends. We can consider the antivirals in the lower right quadrant as widely dispensed but at a lower rate than the previous year. We can consider the upper right quadrant as widely dispensed but at a higher rate than the previous year. The upper right quadrant contains antivirals that are candidates for further evaluation.

A quadrant chart showing the distribution of antiviral agents according to relative rate of change of antibiotic use (2019–2020), and the proportion of antibiotic use in 2020, is presented in Figure 2.

## 3. Discussion

The coronavirus disease 2019 (COVID-19) pandemic has led to growing concern over an increased consumption of antimicrobials for COVID-19 patients, often inappropriately used, and its relation to potentially propagating antimicrobial resistance (AMR) in the short and long term [17,18]. This is particularly an issue in low- and middle-income countries (LMIC) as cost-effective, clinical or biologic markers that effectively discriminate between bacterial and viral infections are lacking [19]. The development of AMR can have a catastrophic impact on health systems in countries with low and middle income levels [20,21]. The results of this study showed a reduction in the use of some antibiotics during the COVID-19 pandemic, for example, penicillins with extended spectrum (amoxicillin) and first-generation cephalosporins. The marked reduction in some antibiotics (e.g., amoxicillin) may be explained by the fact that Jordan was under lockdown during the COVID-19 pandemic. This potentially caused a decrease in person-to-person transmission, possibly decreasing incidence of respiratory tract infections, and fewer patient consultations, e.g., for self-limiting infections that would otherwise have resulted in an antibiotic prescription [22]. Further research is warranted to understand the reasons behind the observed reduction in specific antibiotics. On the other hand, there was increased use of other antibiotics compared with the pre-COVID-19 period (i.e., third-generation cephalosporins, carbapenems, macrolides, and lincosamides). Similar findings were observed in other studies [17,23,24,25]. This increase is of importance since their use has been linked to the development of resistance in several studies [1,4,5,6,26]. It is important to note that carbapenems are used mainly in hospitals and as such the observed increase in use would mainly be in hospitalized patients. The increased use in carbapenems requires further assessment in future work. 

The current study showed an increase in national consumption of azithromycin and hydroxychloroquine during the COVID-19 pandemic study period. This is consistent with other studies that showed an increase in their use in primary care during the COVID-19 pandemic [27,28]. This is not unexpected since both azithromycin and hydroxychloroquine were suggested as potential treatments for hospitalized patients with possible coronavirus 2019 infections (COVID-19) [29]. Azithromycin is a widely available antibiotic with an overall safe profile, which was suggested to have in vitro activity against some viruses, including SARS-CoV-2 [30,31]. Azithromycin has the ability to reduce the levels of proinflammatory cytokines, and thus could reduce the ability to trigger a cytokine storm, along with associated tissue damage by the SARS-CoV-2 infection [32,33]. In addition, azithromycin can be effective in treating co-infection and secondary bacterial infections that might be associated with patients with viral respiratory disease. However, studies showed that azithromycin is not sufficiently effective in treating patients who are admitted to hospital with COVID-19, either alone or in combination with hydroxychloroquine [33,34,35,36]. Of note, the administration of hydroxychloroquine with azithromycin for the treatment of COVID-19-associated pneumonia is associated with an increased risk of cardiac arrhythmias [37]. Thus, the routine use of azithromycin for treatment of COVID-19 in the hospital or in the community is not justified. The increase in azithromycin consumption is concerning since it is associated with risk factors and could contribute to antimicrobial resistance, and thus diminished effectiveness for recommended indications [38]. 

Before the COVID-19 pandemic, hydroxychloroquine was used to treat malaria and rheumatologic conditions. As the coronavirus spread rapidly worldwide, and since it is inexpensive and widely available, hydroxychloroquine was proposed as a treatment for COVID-19. Chloroquine has been demonstrated to have an anti-SARS-CoV activity in vitro [39,40]. Nevertheless, studies showed that hydroxychloroquine is not an effective treatment for hospitalized patients with COVID-19, and the FDA revoked the Emergency Use Authorization (EUA) for chloroquine and hydroxychloroquine and the World Health Organization (WHO) and the National Institutes of Health have ceased trials of its use in hospitalized patients on the grounds of a lack of benefit [41,42,43,44].

The total antibiotic consumption in the pre-COVID-19 and during-COVID-19 periods were 28.4 DDD per 1000 inhabitants per day (96% oral use) and 26.8 DDD per 1000 inhabitants per day (94% oral use), respectively. Data suggest an increase in parenteral antibiotic use administered in hospitalized patients. During 2020, total antimicrobial consumption decreased by 5.5% compared to its consumption in 2019. However, this was associated with marked changes in the use of antibiotics categorized within the WHO Access, Watch, Reserve ‘AWaRe’ classification [7,44,45]. The overall goal should be to reduce the use of Watch and Reserve group antibiotics, and to increase the use of Access group antibiotics where availability is low. As a target and by 2023, 60% of all antibiotics consumed must come from the Access group (https://adoptaware.org/; accessed on 07 June 2021) [7,44,45]. The findings of this study showed that the use of antibiotics in Jordan, in the pre-COVID-19 period, was in line with the recommended WHO targets, i.e., the percentage use of the Access group of national antimicrobial consumption was 59%. However, a marked reduction (by 18%) in the percentage use of Access group (49%) was observed during the pandemic. Use of the Watch group increased by 26% during the pandemic. The use of antibiotics during the current pandemic has undermined efforts to increase use of the Access group and to possibly achieve the WHO target set by 2023. 

This study found that total antiviral use during the COVID-19 pandemic period increased by 37.7% compared to the pre-COVID period. Whereas a marked reduction in some antivirals (acyclovir; −51%) was observed, new antivirals were introduced (favipiravir) as a treatment therapy for COVID-19; favipiravir represented 20% of all antiviral consumption in 2020. In Jordan, a national team for COVID-19 treatments was established at the start of the pandemic, but no protocols were published until October 2020. Favipiravir is not registered in Jordan for COVID-19; however, it has been used since October 2020 for the treatment of COVID-19 patients. Favipiravir has an in vitro inhibitory effect on a range of viruses including coronaviruses, and its use in mild COVID-19 disease was suggested to be associated with clinical benefit relative to other antivirals [46,47,48]. Although, and to date, no resistance has been detected in viruses from favipiravir-treated influenza patients [47], the emergence of antiviral resistance has been documented in other antivirals, e.g., oseltamivir and acyclovir [49,50]. Thus, the emergence of antiviral resistance will result in the need for the development of new antivirals [51].

The study has the strength of using national antimicrobial consumption data covering all antimicrobials delivered to all healthcare settings and community pharmacies. This allowed the assessment of differences between antimicrobial consumption and the AWaRe groups pre- and during the COVID-19 pandemic period. 

The study has some limitations. The study involved the assessment of antimicrobials that were sold in Jordan; it was not possible to assess prescribing practices. In addition, the study would have benefited from separating hospital antimicrobial use from community use which was not possible. Further work is needed to assess differences in antimicrobial use between hospitals and community healthcare settings and pharmacies. In relation to the overall reduction in antibiotic consumption, it was not possible to determine the relative contribution of other factors resulting from the COVID-19 control measures, for example, potential reduction in unnecessary antibiotic use due to reduced healthcare visits and potential reduction in disease transmission. Further work is needed to assess the impact of changes for certain broad spectrum antimicrobials and the increase in Watch group use on the development and spread of antimicrobial resistance. 

Despite these limitations, this study is the first to provide insights about the impact of the COVID-19 pandemic on national antimicrobial consumption in LMICs. This information can help to inform antibiotic stewardship activities in both hospitals and community. Whereas significant work has been done to describe and evaluate antibiotic stewardship in hospitals, experience with outpatient antimicrobial stewardship is limited. Antibiotic stewardship efforts in the community are needed to ensure patient safety and to address potential AMR issues, during and beyond the COVID-19 pandemic. The COVID-19 pandemic has highlighted the severe implications for untreated pathogens, and thus, may offer opportunities to support and implement outpatient antimicrobial stewardship in LMICs. Measuring antimicrobial consumption is an essential strategy for antimicrobial stewardship programs since it allows us to monitor, control, and improve antibiotic use [4,7,13,14].

## 4. Materials and Methods

### 4.1. Settings 

The study involved the collection of national antimicrobial consumption data in hospitals and community pharmacies (population for 2019 = 10,554,000 and 2020 = 10,806,000). Data were obtained from the Jordan Food and Drug Administration (JFDA). JFDA is an independent institution responsible for protecting the Jordanian public health through ensuring the safety, efficacy, and security of medicines and health technologies, as well as ensuring the safety of the food supply reaching consumers in Jordan. These data are collected routinely by JFDA as part of their National Action Plan since 2018. 

### 4.2. Study Design

Antimicrobial consumption data, for the years 2019 and 2020, were collected from JFDA. The collected antimicrobial data represent the combined quantities delivered to Jordanian hospitals and community pharmacies. Using the data from JFDA, it was not possible to separate hospital use from community use. Annual antimicrobial consumption data were collected using an Excel template provided by the WHO [7], which allowed collection of specific relevant data, i.e., antimicrobial agents, dose, strength, quantities, and number of inhabitants in Jordan. The data collected were then validated through an automated process built into the Excel template, as well as through manually checking it for errors. The Excel template contains macros that automatically calculated the DDDs for each antimicrobial agent and population-adjusted consumption estimates, following the classification in the 2019 WHO/Anatomical Therapeutic Chemical (ATC) classification index [7]. DDD is the assumed average maintenance dose per day for a drug used for its main indication in adults. Measuring antibiotic consumption using DDDs, as recommended by the WHO Collaborating Centre for Drug Statistics Methodology, is the most commonly accepted method for reporting antibiotic utilization [7]. The collected data included antibiotics for systemic use (J01), antivirals for systemic use (J05), and antimalarials (P01BA; hydroxychloroquine). Antimicrobial consumption was expressed as DDD per 1000 inhabitants per day. 

### 4.3. Analysis

Descriptive analyses were used to describe antimicrobial consumption. The data were broken down into a graphic containing four quadrants, which were created using the R statistical program and the ggplot2 package [52,53]. The *y*-axis is the relative rate of change of antimicrobial usage from 2019–2020. The *x*-axis is the proportion of the antimicrobial in 2020. The quadrants were created by drawing a vertical line at the average proportion for 2020 and a horizontal line marking the threshold separating positive and negative change in antibiotic use from 2019 levels. The lower left-hand quadrant of the chart represents the antimicrobials that had a negative relative rate of change and a below average proportion of usage in 2020. The upper left-hand quadrant represents the antimicrobials that had positive average relative rate of change and a below average proportion of usage in 2020. The lower right-hand quadrant represents the antimicrobials that had a negative relative rate of change and an above average proportion of usage in 2020. The upper right-hand quadrant represents the antimicrobials that had a positive relative rate of change and an above average proportion of usage in 2020.

Rate of change for each antimicrobial was calculated as (class% 2020/class% 2019). The Drug Utilization 90% (DU90%) indicator aims to focus on the most commonly used drugs, measuring the number of drugs accounting for 90% of the use in DDDs [54]. Antibiotic substances (ATC level 5) were ranked in order of DDDs and the number of drugs accounting for 90% of use is the DU90%. DDD% were calculated by dividing the number of DDDs for a specific antibiotic substance (ATC level 5) by the total DDDs of all antibiotics consumption (J01) and then multiplying by 100%. Antibiotic substances (ATC level 5) were categorized as per the WHO AWaRe classification (Access, Watch, and Reserve) of antimicrobial usage [44].

## 5. Conclusions

In conclusion, the findings of this study provided an assessment of changes in antimicrobial consumption before and during COVID-19 in Jordan. The study highlighted an increase in use of certain antibiotics that are known to be associated with increasing resistance; there was a marked change in antimicrobial use from the Access group towards the Watch group. Efforts to monitor and enhance antimicrobial stewardship activities at the national level are needed to ensure patient safety and the appropriate use of antimicrobials. Future directions for rationalising antibiotic use by JFDA include the following: to enforce implementation of antibiotics with prescription only from pharmacies, to adapt AWaRe classification on registered antibiotics, to monitor the clinical practice of prescribing and dispensing of antibiotics, and to undertake education and awareness campaigns and activities. Good prescribing practices, even during a pandemic, will need to be maintained to prevent future issues with resistant bacteria.

## Figures and Tables

**Table 1:** National antimicrobial consumption, expressed in DDD per 1000 inhabitants per day per antimicrobial class, for 2019 and 2020, Jordan.

Antimicrobial Class	National Antibiotic Use 2019		National Antibiotic Use 2020		Relative Rate of Change *
	Total DDD per 1000 Inhabitants per Day	Class%	Total DDD per 1000 Inhabitants per Day	Class%	
Tetracyclines	1.508	5.312%	1.5673	5.844%	1.10
Penicillins with extended spectrum	8.396	29.574%	3.7361	13.930%	0.47
Beta-lactamase resistant penicillins	0.002	0.008%	0.0024	0.009%	1.06
Combinations of penicillins, including beta-lactamase inhibitors	4.843	17.059%	4.8462	18.069%	1.06
First-generation cephalosporins	1.147	4.042%	0.7015	2.616%	0.65
Second-generation cephalosporins	2.650	9.335%	2.5534	9.520%	1.02
Third-generation cephalosporins	2.024	7.128%	2.2750	8.482%	1.19
Fourth-generation cephalosporins	0.000	0.001%	0.0023	0.009%	9.35
Carbapenems	0.043	0.152%	0.0618	0.230%	1.52
Other cephalosporins and penems	0.001	0.003%	0.0003	0.001%	0.34
Combinations of sulfonamides and trimethoprim, including derivatives	0.170	0.598%	0.3646	1.359%	2.27
Macrolides	3.238	11.405%	4.8133	17.947%	1.57
Lincosamides	0.714	2.514%	1.3892	5.180%	2.06
Other aminoglycosides	0.046	0.162%	0.0425	0.158%	0.98
Fluoroquinolones	2.745	9.669%	3.0007	11.188%	1.16
Combinations of antibacterials	0.416	1.464%	0.5483	2.044%	1.40
Glycopeptide antibacterials	0.046	0.162%	0.0429	0.160%	0.98
Polymyxins	0.003	0.010%	0.0025	0.009%	0.90
Imidazole derivatives	0.394	1.389%	0.8657	3.228%	2.32
Other antibacterials	0.004	0.013%	0.0028	0.010%	0.81

* Relative rate of change is calculated as (class% 2020/class% 2019).

**Table 2:** Percentage of total national antimicrobial consumption (DDDs) by WHO AWaRe category (Access/Watch/Reserve) for Jordan 2019 and 2020.

Year	Access	Watch	Reserve
	DDD per 1000 Inhabitants per Day (%)		
National antimicrobial use (2019)	16.79 (59.13)	11.59 (40.83)	0.011 (0.0376)
National antimicrobial use (2020)	13.05 (48.67)	13.76 (51.30)	0.008 (0.0315)
Relative rate of change *	0.82	1.26	0.84

* Relative rate of change is calculated as (DDD per 1000 inhabitants per day% 2020/DDD per 1000 inhabitants per day% 2019).

**Table 3:** National antimicrobial consumption, contributing to 90% of total national antibiotic use in 2019 and 2020, and categorized by WHO AWaRe category (Access/Reserve/Watch).

National Antimicrobial Use (2019)			National Antimicrobial Use (2020)		
Antimicrobials *	DID **	DIDs% **	Antimicrobials *	DID **	DIDs% **
Amoxicillin	8.39	29.56%	Amoxicillin/clavulanic Acid	4.83	17.99%
Amoxicillin/clavulanic Acid	4.82	16.97%	Amoxicillin	3.73	13.91%
Cefuroxime	2.37	8.34%	Azithromycin	2.55	9.49%
Azithromycin	1.54	5.44%	Cefuroxime	2.31	8.60%
Ciprofloxacin	1.53	5.40%	Clarithromycin	1.87	6.98%
Doxycycline	1.38	4.87%	Ciprofloxacin	1.85	6.89%
Cefixime	1.25	4.40%	Cefixime	1.51	5.63%
Clarithromycin	1.21	4.26%	Doxycycline	1.32	4.94%
Cefalexin	1.11	3.90%	Clindamycin	1.19	4.43%
Levofloxacin	1.02	3.58%	Levofloxacin	0.89	3.32%
Clindamycin	0.43	1.52%	Metronidazole (IV)	0.87	3.23%
Spiramycin/metronidazole	0.42	1.46%	Cefalexin	0.61	2.28%
Metronidazole (IV)	0.39	1.39%	Spiramycin/metronidazole	0.55	2.04%
			Sulfamethoxazole/trimethoprim	0.36	1.36%

* Colour code: Green = Access, amber = Watch. ** DID: DDD per 1000 inhabitants per day.

**Table 4:** National antivirals consumption, expressed in DID (DDD/1000 inhabitants/days) per each agent, for 2019 and 2020, Jordan.

Antiviral Agent	National Antibiotic Use 2019		National Antibiotic Use 2020		Relative Rate of Change *
	Total DDD per 1000 Inhabitants per Day	Class%	Total DDD per 1000 Inhabitants per Day	Class%	
Aciclovir	0.058	38.87%	0.039	18.99%	0.49
Ganciclovir	0.000	0.31%	0.000	0.16%	0.51
Valaciclovir	0.014	9.12%	0.011	5.40%	0.59
Valganciclovir	0.001	0.94%	0.001	0.70%	0.75
Lamivudine	0.014	9.32%	0.037	18.01%	1.93
Adefovir dipivoxil	0.003	1.98%	0.001	0.73%	0.37
Entecavir	0.038	25.37%	0.044	21.57%	0.85
Tenofovir alafenamide	0.001	0.39%	0.002	0.92%	2.34
Oseltamivir	0.020	13.70%	0.029	13.95%	1.02
Favipiravir	0.000	0.00%	0.040	19.56%	NA

* Relative rate of change is calculated as (class% 2020/class% 2019).

## Data Availability

Data available on reasonable request and in line with permission approval processes from the JFDA.
